# Complete Heart Block in a Patient Declining Pacemaker Implantation: A Discussion on Patient-Centered Care and Shared Decision-Making

**DOI:** 10.7759/cureus.75241

**Published:** 2024-12-06

**Authors:** Robert W Buntyn, Vraj Patel, Hope N Lewis, Thein T Aung, Vaskar Mukerji

**Affiliations:** 1 Internal Medicine, Wright State University Boonshoft School of Medicine, Dayton, USA; 2 Cardiology, Wright State University Boonshoft School of Medicine, Dayton, USA

**Keywords:** av node, complete heart block, decision-making autonomy, first-degree heart block, goals of care, pacemaker, patient-centered care, second-degree heart block

## Abstract

Permanent pacemaker (PPM) implantation is the standard of care in patients with complete heart block (CHB) and second-degree type II atrioventricular (AV) block irrespective of patient symptoms when the conduction abnormality is irreversible. CHB generally constitutes a medical emergency that can be fatal if not urgently treated. This is in contrast to first-degree AV block and second-degree type I AV block, which require PPM implantation only in very special circumstances. While second-degree type II AV block is considered to be at high risk for progression to CHB, this does not occur with first-degree and second-degree type I AV blocks.

Here, we present a patient who demonstrated over a number of years a progression from first-degree to second-degree type I AV block and then, quite unexpectedly, to CHB. Despite multiple discussions of the benefits of PPM implantation with cardiology staff, the patient elected to forgo the procedure. This case discusses patient-centered care and the therapeutic dilemma that can develop when there is an incongruence between the principles of beneficence and patient autonomy.

## Introduction

Complete heart block (CHB) is the complete loss of electrical conduction between the sinus node in the atria and the ventricles. The conduction abnormality may be within the atrioventricular (AV) node itself or in an infranodal location. Symptoms and death may occur when the resultant escape rhythm is unable to maintain a sustainable cardiac output. Permanent pacemaker (PPM) implantation is the standard of care in patients with CHB and second-degree type II AV block when the conduction abnormality is irreversible. This recommendation remains even in the absence of symptoms. Failure to treat this condition with PPM implantation can be fatal [1,2].

Patient-centered care, which is an emphasis on an individual’s health needs for healthcare decisions, is considered important in patients with multimorbidity [3]. In these complex patients, the benefits and risks of interventions extrapolated from population-derived statistics and strictly controlled clinical trials may not be entirely accurate [4]. Infection, bleeding, pneumothorax, and device system failure are complications associated with PPM implantation. Although these risks are low, the mortality benefit of a PPM generally outweighs the risk.

Here, we present a patient who over several years demonstrated progression from first-degree to second-degree type I AV block and then, surprisingly, CHB but declined PPM placement.

## Case presentation

An 82-year-old man with a past medical history of three-vessel coronary artery bypass surgery 30 years ago, left ventricular ejection fraction of 40%, hypertension, diabetes mellitus, chronic kidney disease, peripheral vascular disease, and deep venous thrombosis presented to the hospital with melena. This patient was wheelchair-bound at baseline, and he required assistance with most of the activities of daily living. He denied cardiac symptoms.

**Figure 1:**
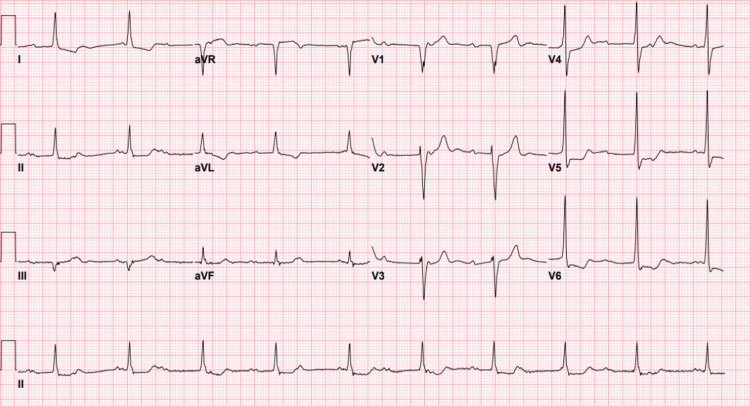
Sinus rhythm and third-degree AV block (2023) AV: atrioventricular

**Figure 2:**
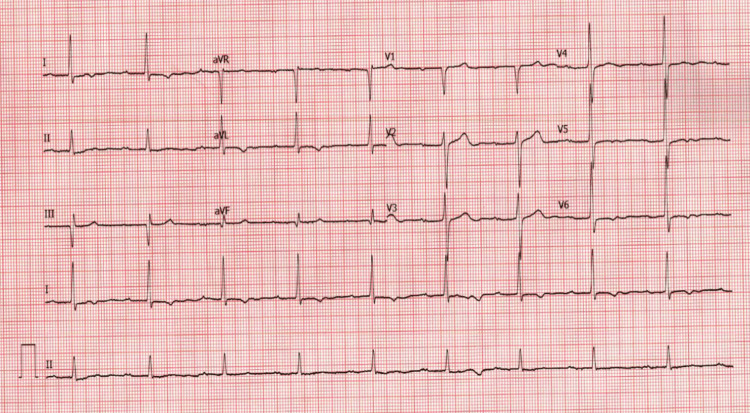
Sinus bradycardia and first-degree AV block (2016) AV: atrioventricular

**Figure 3:**
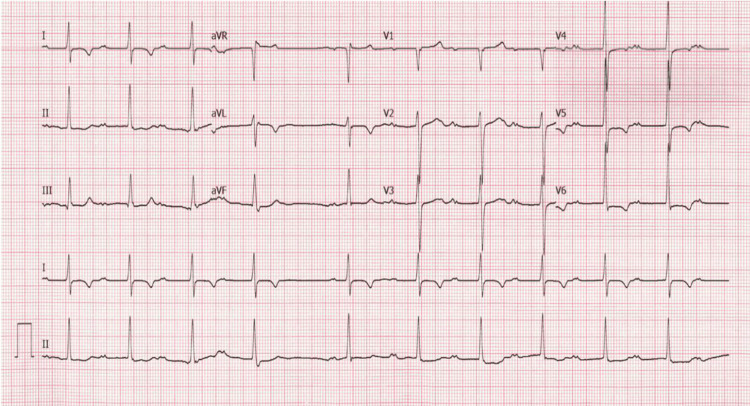
Sinus rhythm and second-degree type I AV block (2018) AV: atrioventricular

**Figure 4:**
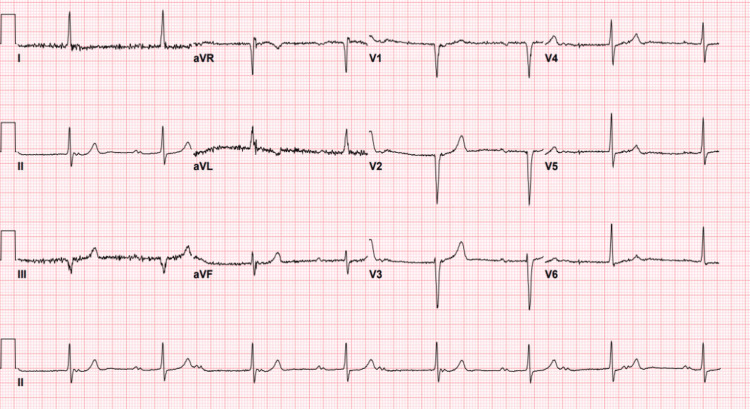
Sinus rhythm and third-degree AV block (early 2022) AV: atrioventricular

His labs were significant for a hemoglobin of 6.4 g/dL (13.0-17.7 g/dL), a platelet count of 107 K/uL (140-400 K/uL), a blood urea nitrogen (BUN) of 32 mg/dL (3-29 mg/dL), potassium of 5.0 mEq/L (3.4-5.3 mEq/L), and a creatinine of 2.3 mg/dL (0.5-1.4 mg/dL). Electrocardiogram (ECG) showed CHB (Figure 1) for which the cardiology service was consulted. The ventricular rate during CHB ranged from 47 to 59 bpm with a narrow QRS complex. Previous ECGs over a number of years showed progression from first-degree heart block to second-degree type I and finally to CHB (Figures 2-4). The patient was not taking AV nodal-blocking agents. He was transfused with two units of packed red blood cells, and his hemoglobin improved to 9.1 g/dL. A PPM was offered, but the patient declined. Upper endoscopy was offered, but the patient also declined, and he was empirically treated for peptic ulcer disease. He had no significant symptoms following his blood transfusions, and he was discharged home.

## Discussion

First-degree AV block and second-degree type I AV block are generally considered to be benign abnormalities. They carry a low risk of progression to a higher degree of AV block. The Framingham study followed 124 patients with first-degree AV block; only four demonstrated progression to second-degree AV block. There were no patients within that population who developed CHB [5]. In the United States, we lack data for untreated CHB. A prospective study in Cameroon noted a mortality of 45% in patients with untreated CHB during their study period of six years [6]. Another study in Togo noted a 59% mortality rate with untreated CHB over five years [7].

Under current guidelines, PPM implantation is recommended for all patients with CHB and second-degree type II heart block irrespective of symptoms if no reversible cause is identified [1]. However, this patient presented a situation that was unique. He was admitted to the hospital with gastrointestinal bleeding for which he was treated, and his presenting symptoms resolved. The CHB with a narrow QRS complex was an incidental finding for which he reported no symptoms. Further review of ECGs revealed that this arrhythmia had been present for a number of years without intervention. Despite multiple risk and benefit discussions with this patient, he continued to decline PPM implantation.

Patient-centered care is a focus on medical decision-making with consideration for a patient’s preferences, beliefs, and values [8]. As physicians, we often find ourselves focused on offering treatments or procedures for conditions like CHB without consideration of alternatives. Under the presumption that an acceptable outcome for CHB is an improvement in symptoms and a reduction in mortality, PPM implantation is the treatment of choice. A dilemma occurs for physicians when a patient declines the recommended treatment. How does one weigh the principles of beneficence and autonomy?

The American Medical Association defines beneficence as the ethical obligation of a physician to act in the best interest of the patient, whereas autonomy is defined as the right of patients with the capacity to make informed decisions about their medical care [9]. Many theories of medical ethics have set out to reconcile scenarios where these two principles may disagree. One theory is that patient benefit can have two different definitions: one that focuses on the patient’s view of the good and another that focuses on an objective view of health, which is the physician's responsibility to promote [10]. In this case, the patient was asymptomatic and therefore viewed it as beneficial to avoid a procedure that could cause him harm. Objectively, the ideal health benefit would be the guideline-directed placement of a PPM for improved mortality and quality of life outcomes, which this patient did not agree to pursue. This dilemma can be reconciled by defining a successful outcome as an outcome that is meaningful and valuable to the patient.

Our patient had multiple comorbidities, which could offer a worse prognosis than CHB. He had a limited functional status at baseline. His heart rate appeared to support his limited lifestyle, and he reported no related symptoms. It was unclear whether PPM implantation would have any impact on his overall prognosis or quality of life. The reasons why this patient declined PPM implantation are unclear; however, he had been declining procedural interventions for other health conditions over the previous years. Perhaps this patient considered the avoidance of a procedure or risk of procedural complication as more valuable than treatment of a condition that caused him no symptoms.

To our knowledge, no case reports have discussed the implications of progressive conduction disease, asymptomatic CHB, and declination of a PPM. A different scenario is represented in the literature, which includes the appropriate timing of device placement in a younger individual with asymptomatic CHB. In these circumstances, the resultant escape rhythm would eventually cause symptoms, but placement of a PPM too early may expose patients to unnecessary procedural complications without therapeutic benefit [11]. Conversely, in a patient with complex multimorbidity, the placement of a PPM presents a different therapeutic dilemma: does the PPM prolong suffering from the patient’s other medical conditions? Prudent discussion of the patient’s goals and values when discussing medical interventions can help navigate these scenarios.

Decision aids are tools designed to facilitate the decision-making process between patients and physicians. These aids can be present in a number of modalities, including web-based information, pamphlets, and apps. The information in these aids presents the risks and benefits of treatment options in a manner that educates patients and supports informed decisions. People who use decision aids report feeling more knowledgeable and better informed [12]. Our patient was presented with a standard brochure about PPM implantation during an outpatient cardiology visit when CHB was first identified, but it is unclear if this was a decision aid or informational. The National Quality Forum is a United States nonpartisan organization that has determined the need for a certification process for decision aids, but no decision aid currently exists for the placement of PPM in this scenario.

When a physician and patient are approaching a difficult decision in guiding medical care, shared decision-making is the best course of action. Exercising shared decision-making has the potential to not only guide medical care to be in line with patient values but also improve patient understanding, satisfaction, healthcare costs, and outcomes [13,14]. This patient ultimately made an informed decision not to proceed with PPM implantation despite his diagnosis. Perhaps the medically defined benefits of a PPM were neither meaningful nor valuable for this patient.

## Conclusions

Untreated CHB is an uncommon occurrence in the United States, and the situations where it may occur are limited. This patient presented with progressive conduction disease from first-degree AV block to CHB. The slowly progressive AV node block, acceptable escape rate for this patient's functional status, multimorbidity, and patient preferences created a situation with no clearly correct medical decision.

Patient-centered care is a critical component in the delivery of high-quality healthcare. This case presented a circumstance where the patient's preference significantly altered care with an overall unexpected outcome. While medicine has guidelines and standards of care for the management of various medical conditions, physicians must also consider the preferences, beliefs, and values of their patients.
